# APHS-YOLO: A Lightweight Model for Real-Time Detection and Classification of Stropharia Rugoso-Annulata

**DOI:** 10.3390/foods13111710

**Published:** 2024-05-29

**Authors:** Ren-Ming Liu, Wen-Hao Su

**Affiliations:** College of Engineering, China Agricultural University, Haidian, Beijing 100083, China; buchuanneiku168@gmail.com

**Keywords:** Stropharia rugoso-annulata, automatic sorting, high-level screen feature pyramid, lightweight model, knowledge distill

## Abstract

The classification of Stropharia rugoso-annulata is currently reliant on manual sorting, which may be subject to bias. To improve the sorting efficiency, automated sorting equipment could be used instead. However, sorting naked mushrooms in real time remains a challenging task due to the difficulty of accurately identifying, locating and sorting large quantities of them simultaneously. Models must be deployable on resource-limited devices, making it challenging to achieve both a high accuracy and speed. This paper proposes the APHS-YOLO (YOLOv8n integrated with AKConv, CSPPC and HSFPN modules) model, which is lightweight and efficient, for identifying Stropharia rugoso-annulata of different grades and seasons. This study includes a complete dataset of runners of different grades in spring and autumn. To enhance feature extraction and maintain the recognition accuracy, the new multi-module APHS-YOLO uses HSFPNs (High-Level Screening Feature Pyramid Networks) as a thin-neck structure. It combines an improved lightweight PConv (Partial Convolution)-based convolutional module, CSPPC (Integration of Cross-Stage Partial Networks and Partial Convolution), with the Arbitrary Kernel Convolution (AKConv) module. Additionally, to compensate for the accuracy loss due to lightweighting, APHS-YOLO employs a knowledge refinement technique during training. Compared to the original model, the optimized APHS-YOLO model uses 57.8% less memory and 62.5% fewer computational resources. It has an FPS (frames per second) of over 100 and even achieves 0.1% better accuracy metrics than the original model. These research results provide a valuable reference for the development of automatic sorting equipment for forest farmers.

## 1. Introduction

Improving the grading of Stropharia rugoso-annulata is important for enhancing its competitiveness in the market. Verma et al. [1] suggest that manual grading may be labor-intensive, inefficient, and inconsistent. Furthermore, the popularity of Stropharia rugoso-annulata in China, due to its medicinal and culinary benefits, has led to an increase in production [2]. However, it is important to acknowledge that the current method of sorting Stropharia rugoso-annulata is mainly manual, which presents a challenge to the industrialization of the forest economy and reduces production efficiency. Therefore, it would be advisable to develop a real-time and intelligent algorithm for detecting and grading Stropharia rugoso-annulata. To ensure reliable performance, it is essential to establish a comprehensive grading dataset for Stropharia rugoso-annulata.

Various methods have been proposed for the intelligent classification of mushrooms based on spectral analysis and deep learning techniques for machine vision. However, critics have pointed out issues with the representativeness and completeness of the dataset, as well as the simple preprocessing, outdated deep learning models, and low recognition efficiency. In 2022, Chen, Yan, Xiong et al. [3] proposed a lightweight improved machine vision detection algorithm based on YOLOv4. The model achieved an accuracy of 90.63% and a speed improvement of 150%. However, it is important to note that the model was trained on a smaller dataset and used an outdated version of the YOLOv4 framework. Wang, Feng, and Zheng [4] proposed a double-hugging mushroom image size classification algorithm based on watersheds, Canny operators, and closure operations. The algorithm achieved an accuracy of 97.42%. However, the algorithm does not consider model lightweighting and is therefore unsuitable for resource-constrained devices. Chen and Ting [5] proposed an automatic algorithm for grading mushrooms based on their shape, color and size. The algorithm used image processing techniques, such as color charge-coupled cameras and geometric feature extraction, to identify features of shiitake mushrooms, including abnormal colors and damaged caps. However, the method lacks quantitative metrics. To address this issue, Huang et al. [6] utilized computer vision techniques to extract seven shape-related feature parameters for identifying defects in broken straw mushrooms. These parameters include the fractal dimension, relative length, roundness, shape factor, convexity, aspect ratio, and concavity of the gills, as well as the curvature, aspect ratio, and stipe of the gills. The authors developed an SVM (support vector machine) model to differentiate mushrooms from different classes. However, describing the geometric and morphological features of Stropharia rugoso-annulata using traditional methods can be complex and time-consuming. In recent years, researchers have successfully classified and recognized Stropharia rugoso-annulata using deep learning methods. These methods are less labor-intensive and have the potential to advance the mushroom industry. The quick and accurate classification of mushroom images using deep learning technology is significant. For example, Liu et al. [7] proposed an improved YOLOX deep learning method with an efficient channel pruning mechanism for mushroom grading, which effectively detects the surface texture of mushrooms. The XCA-MobileViT model, developed by Zuo, Zhao, Wu and Li [8], achieved an mAP (mean average precision) of 99 and an FPS (frame per second) of 99. It includes a multiscale module that enhances the fusion of local and global features and a dual-attention module for classifying Stropharia rugoso-annulata. The model achieved an average recognition accuracy of 97.71%, which is 2.34% higher than that of the MobileViT model.

Most existing research focuses on improving model accuracy, but few studies consider lightweighting models or both. However, in practice, high-accuracy models often require more resources, while lightweight models may sacrifice recognition accuracy. Additionally, there is no research on the issue of the detection and recognition speed, which is a key factor to consider when classifying a large number of mushrooms simultaneously. Therefore, this study proposes a lightweight model, APHS-YOLO, that balances the recognition accuracy, speed, and computational resources by employing knowledge refinement techniques during training. The materials and methods used in this study, as well as the details of the experiments conducted and the results obtained, along with limitations and future perspectives, are presented sequentially in Section 2, Section 3 and Section 4, and are used together to substantiate the conclusions presented.

## 2. Materials and Methods

This section will start by explaining how we obtained the Stropharia rugoso-annulata dataset for the experiment. Then, we will introduce the model and provide a detailed description of its innovations.

### 2.1. Dataset Acquisition and Image Enhancement

**Figure 1:**
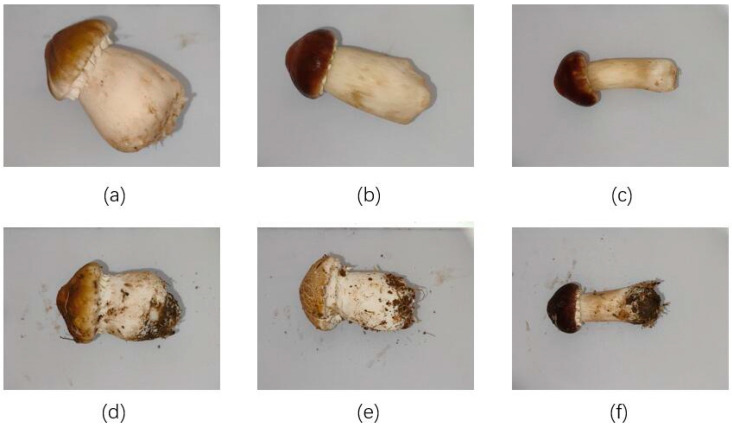
Classification of Stropharia rugoso-annulata: (**a**) Autumn first class. (**b**) Autumn second class. (**c**) Autumn third class. (**d**) Spring first class. (**e**) Spring second class. (**f**) Spring third class.

**Figure 2:**
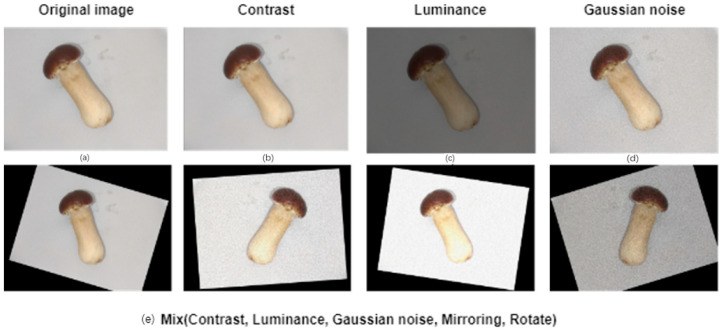
Original and enhanced images: (**a**) Original. (**b**) Changed contrast. (**c**) Changed brightness. (**d**) Gaussian noise added. (**e**) All means of image enhancement mixed.

This paper focuses on Stropharia rugoso-annulata, and the accompanying images were obtained from the experimental base of the Research Institute of Pinggu District, Beijing. The images were captured using an MV-UBD130C industrial camera (MindVision, Shenzhen, China) with a spatial resolution of 1280 × 960, a frame rate of 35 FPS, a 4-megapixel lens, a 6 mm focal length, and a 20 cm distance from the target, taken between 12:00 and 16:00 h. A total of 2032 images of Stropharia rugoso-annulata, of varying grades, were collected during the spring and fall under identical light and background conditions. These images were then divided into a training set, validation set, and test set in an 8:1:1 ratio. Table 1 outlines the classification rules for heather, while Figure 1 displays images of heather of different grades during both seasons. The images were labeled manually using the image annotation tool ‘Labelme 4.5.13’, and the groups of the same grade images were labeled using external rectangular boxes. To avoid overfitting problems caused by an insufficient number of images in the dataset and to improve the generalization ability and robustness of the deep learning network, a total of 6490 images were obtained by rotating, adding noise, adjusting brightness, and performing other image enhancement operations on the original images in each dataset. The dataset was enhanced for network model training, as shown in Table 2. The effect of the enhancement is illustrated in Figure 2.

### 2.2. The Network Structure of APHS-YOLO

**Figure 3:**
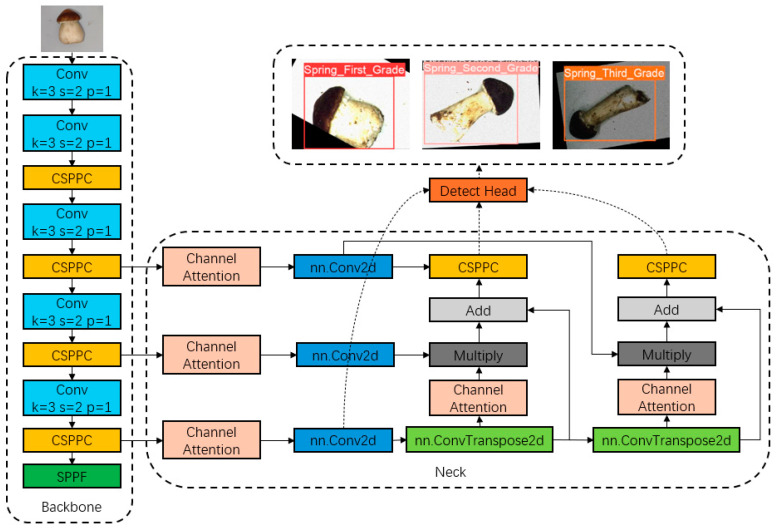
Representation of the structure of APHS-YOLO.

Figure 3 shows the network structure of the YOLOv8 improved version of APHS-YOLO and its prediction results. The image size of the input network is 640 × 640, and the APHS-YOLO network model designed by us gives the accurate recognition results of three classes of Stropharia rugoso-annulata. The torso of the model uses our self-developed CSPPC lightweight convolution module, AKConv module, HSFPN module, etc., which enable the network to reduce the redundant parameters without affecting the accuracy, to effectively pass the feature information downwards, and to improve the operation speed.

In general, the lightweight modules (CSPPC, AKConv, HSFPN) reduce the size of the model and increase the speed of detection, so that the model can be mounted without relying on expensive equipment, which reduces the cost of the equipment and the waste of computational resources. The attention module (CGA and ACmix) increases the accuracy of the model in detecting Stropharia rugoso-annulata grades. The combination of these two modules in APHS-YOLO enables the APHS-YOLO model to be mounted on a simple device for highly accurate and fast grade detection of Stropharia rugoso-annulata.

#### 2.2.1. CSPPC (Integration of Cross-Stage Partial Networks and Partial Convolution) Lightweight Module

This study proposes a CSPPC module that combines the lightweight concept of double convolution with the structure of Partial Convolution [9] (PConv). The memory access of PConv is shown in Equation (1).

(1) $$h\times w\times 2c_{p}+k^{2}\times c_{p}^{2}\approx h\times w\times 2c_{p}$$

**Figure A1:**
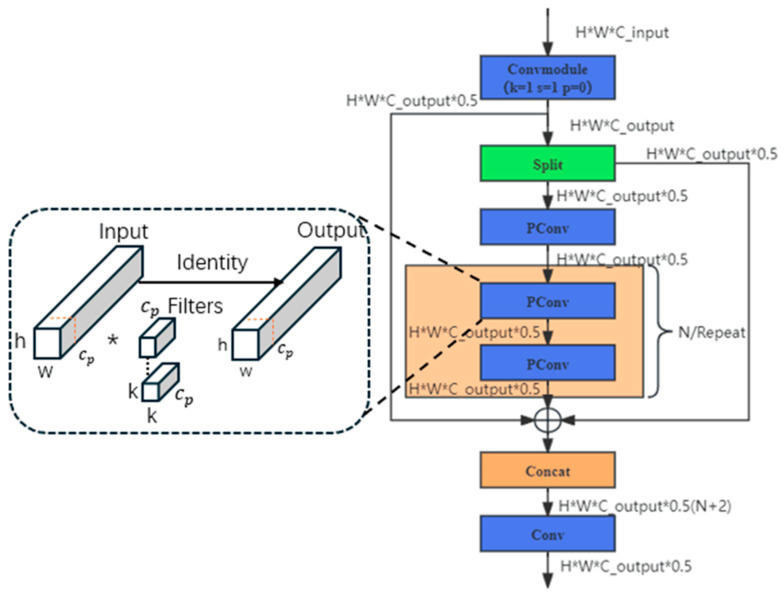
Structure diagram of CSPPC.

The CSPPC module, a lightweight innovative module based on the PConv proposal, replaces C2f and is integrated into the backbone network of the algorithm. This integration eliminates highly similar channel features, reduces computational redundancy and memory accesses, reduces the number of parameters and improves detection speed. The module connects two PConvs in series during the output process, reducing the amount of computational parameters of the model used for the experiment by more than half a million. Figure A1 illustrates the structure of the CSPPC module. CSPPC can significantly reduce the size of the model for easy piggybacking, which in turn reduces the cost of grading equipment development.

#### 2.2.2. Arbitrary Kernel Convolution (AKConv) Lightweight Module

**Figure A2:**
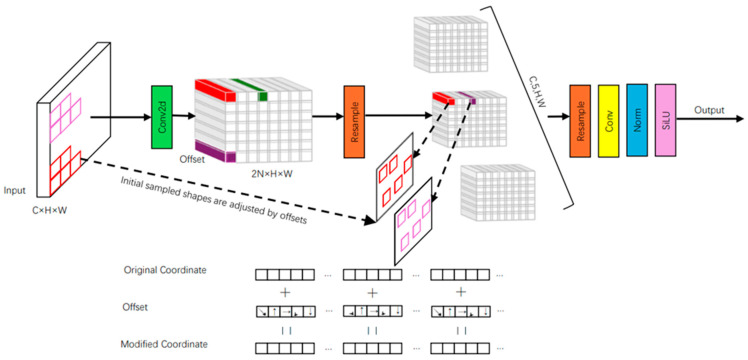
Structure diagram of AKConv (The arrows in Offset indicate the direction of movement of the sampling coordinates).

AKConv [10] endows the convolution kernel with an arbitrary number of parameters and arbitrary sample shapes, providing richer options for the trade-off between network overhead and performance. That is, it allows the network to simplify the Stropharia rugoso-annulata images as appropriate when identifying Stropharia rugoso-annulata grades, resulting in more detailed and rich features of Stropharia rugoso-annulata, which helps to optimize the performance in hardware environments. Figure A2 shows the network structure and workflow of AKConv.

#### 2.2.3. High-Level Screening Feature Pyramid Networks (HSFPNs)

**Figure A3:**
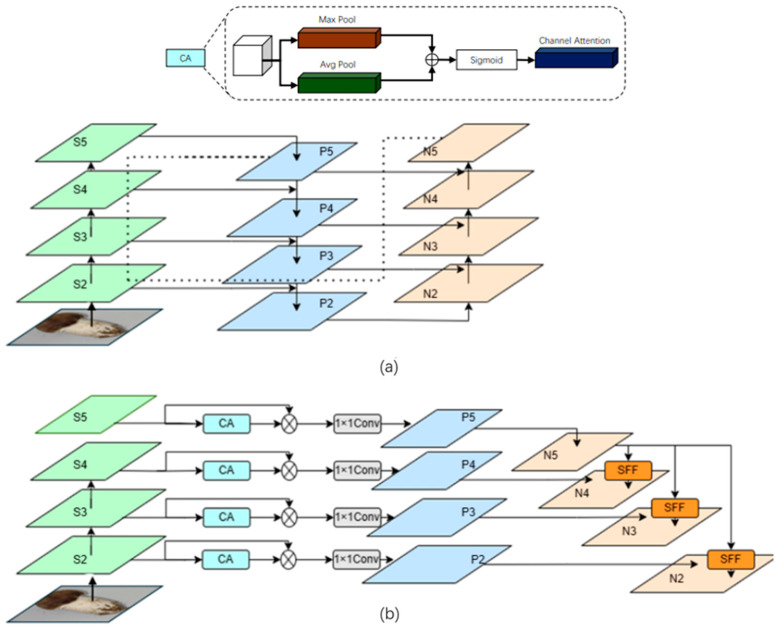
Comparison of neck network structure: (**a**) PAN, (**b**) HSFPN (The dotted line indicates that the output of S2 is used as an input to P5 and N5).

HSFPN is an architecture proposed by the Improvement Mechanism Multi-Level Feature Fusion and Deformable Self-Attention DETR (MFDS-DETR) [11]. It consists of two key components: a feature selection module and a feature fusion module. The Channel Attention (CA) and Dimensional Matching (DM) in the feature selection module match feature maps at different scales. Figure A3 depicts the schematic diagram of the HSFPN module.

In brief, the Stropharia rugoso-annulata images put into the HSFPN are processed into three sizes, large, medium and small, before being detected in the hierarchy, and then each of them is filtered and extracted to process the parts of them that are only related to Stropharia rugoso-annulata, and finally, the results of these processes are unified into one dimension and then outputted to the next layer of the network. Such processing allows the APHS-YOLO model to recognize Stropharia rugoso-annulata without being influenced by factors that are not related to Stropharia rugoso-annulata, and is more objective compared to manual classification.

The HSFPN in APHS-YOLO shows a significant lightweighting effect, reducing the model size by 35%, with one million parameters from the original model, which makes it possible to mount Stropharia rugoso-annulata on resource-limited devices and then perform the identification task.

### 2.3. Attention Mechanism

#### 2.3.1. Cascaded Group Attention (CGA) Module

The traditional transformer utilizes a self-attention mechanism that processes all pixels in an image, resulting in a significant computational load. In contrast, the Cascaded Group Attention [12] (CGA) method processes the same image of Stropharia rugoso-annulata in different groups for computational processing, where each group focuses on a different aspect of the Stropharia rugoso-annulata features, thus enhancing the diversity of the attention map. This optimization improves computational efficiency and memory usage, while maintaining or even improving the performance of the model.

#### 2.3.2. ACmix (Integration of Self-Attention and Convolution) Module

ACmix [13] is a hybrid model that combines the advantages of the self-attention mechanism and convolutional operations. ACmix can reduce the attention to invalid information and focus only on the global features related to Stropharia rugoso-annulata when processing Stropharia rugoso-annulata images, and also selectively captures the local features of Stropharia rugoso-annulata, which achieves a reduction in the processing time and speeds up the detection.

### 2.4. Evaluation Metrics

In order to demonstrate the rigor of this study and make the comparison of the data more credible and valuable, the evaluation of APHS-YOLO used well-established assessment metrics, which include precision (P), mean average precision (mAP), recall (R), and frames per second (FPS). These metrics have been similarly used in studies such as PASCAL VOC [14] and MS COCO [15], which reflects the validity and generalizability of the chosen evaluation metrics. In addition, in order to better reflect the degree of model lightweighting, we have used metrics such as the number of parameters (parameter), floating point operations (GFLOPs), and the amount of memory occupied by the model. The relevant formulas are as follows:

Precision (*P*) indicates how many of the samples with positive predictions are correct (Equation (2)):

(2) $$P=\frac{TP}{TP+FP}$$

Recall (*R*) indicates the number of samples that are predicted to be positive out of those that are truly positive, as in Equation (3).

(3) $$R=\frac{TP}{TP+FN}$$

Mean average precision (*mAP*) is used to calculate the average precision (*AP*) of multiple categories, as denoted in Equation (5). It is an averaging process for the precision of each category, as defined in Equation (4):

(4) $$mAP=\frac{1}{n}\sum_{k=1}^{n}AP_{k}$$

(5) $$AP_{k}=\int_{0}^{1}p\left(r\right)dr$$

where *n* denotes the number of categories and $AP_{k}$ denotes the accuracy of the *k*th category. True Positive (*TP*) means that the prediction is positive and the labeled value is also positive and the prediction is correct; False Negative (*FN*) means that the prediction is negative and the labeled value is positive and the prediction is incorrect; False Positive (*FP*) means that the prediction is positive and the labeled value is negative and the prediction is incorrect; True Negative (*TN*) indicates that a negative case is predicted, the labeled value is negative, and the prediction is correct.

## 3. Experiment

### 3.1. Experiment Environment Setting

The PyTorch 1.11.0 deep learning framework was used in this study. The experiments were conducted on an Ubuntu 20.04 Cuda 11.3 operating system, and trained and validated on NVIDIA RTX 4090 24G GPUs (Santa Clara, CA, USA). The experimental hyperparameters are shown in Table 3.

### 3.2. Model Selection

YOLOv8 is a cutting-edge, state-of-the-art (SOTA) model. It can perform a variety of object detection tasks quickly, accurately and easily. Different models are provided for different application scenarios, including YOLOv8n, YOLOv8s, YOLOv8l, YOLOv8x and YOLOv8m, which differ only in depth and width. In practice, Stropharia rugoso-annulata are graded in order to achieve just-in-time sorting, and therefore, the model sizes are required to be sufficiently small and the detection speeds are sufficiently fast to be able to adapt to the deployment of intelligent machinery. Therefore, after comprehensive consideration, YOLOv8n was finally selected as the base model for the student model and YOLOv8s as the base model for the teacher model. Comparison of the training results for different models of YOLOv8 is shown in Table 4.

### 3.3. Knowledge Distillation Experiment

It is difficult for models to be practically applied with resource-constrained devices due to the huge amount of computation and number of parameters [16]. In order to make deep models more efficient, knowledge distillation is one of the research directions. Hinton [17] first introduced the concept of knowledge distillation and introduced temperature coefficients to manipulate it. Knowledge distillation can be used as a method of model acceleration and compression.

To address the issue of reduced accuracy in lightweight experiments, this study conducted knowledge distillation-assisted training experiments. The APHS-YOLO model, after undergoing knowledge distillation, achieved fast convergence and high accuracy while maintaining its lightweight nature. The objective function of high-temperature distillation is specifically obtained by the weighted sum of distillation loss, which corresponds to the soft target, and student loss, which corresponds to the hard target. This is shown in Equation (6). The first part, $L_{soft}$, is the cross-entropy of the softmax distribution produced by the teacher model as a soft target at high temperature and the softmax output produced by the student model at the same temperature, as shown in Equation (7). The second part, $L_{hard}$, is the cross-entropy between the softmax output of the student model at *T* = 1 and the ground truth, as shown in Equation (8).

(6) $$L=\alpha_{2}L_{soft}+\beta_{2}L_{hard}$$

(7) $$L_{soft}=-\sum_{j}^{N}p_{j}^{T}\log\left(q_{j}^{T}\right),\left(p_{j}^{T}=\frac{\exp\left(\frac{v_{i}}{T}\right)}{\sum_{k}^{N}\exp\left(\frac{v_{k}}{T}\right)},q_{i}^{T}=\frac{\exp\left(\frac{z_{i}}{T}\right)}{\sum_{k}^{N}\exp\left(\frac{z_{k}}{T}\right)}\right)$$

(8) $$L_{hard}=-\sum_{j}^{N}c_{i}\log\left(q_{j}^{1}\right),\left(q_{i}^{1}=\frac{\exp\left(z_{i}\right)}{\sum_{k}^{N}\exp\left(z_{k}\right)}\right)$$

where $v_{i}$ is the logits of teacher model, $z_{i}$ is the logits of student model, $p_{j}^{T}$ is teacher model’s softmax output at temperature *T* on the value of class *i*, $q_{i}^{T}$ is student model’s softmax output at temperature *T* on the value of class *i*, $c_{i}$ is the ground truth value on class *i*, *N* is the total number of labels.

For the formal experiments, we chose our proposed APHS-YOLO model as the student model and the improved YOLOv8s model with the CGA module, ACmix module and HSFPN structure as the teacher model. We used the high accuracy of the teacher model to assist in training the highly lightweight student model.

**Figure 4:**
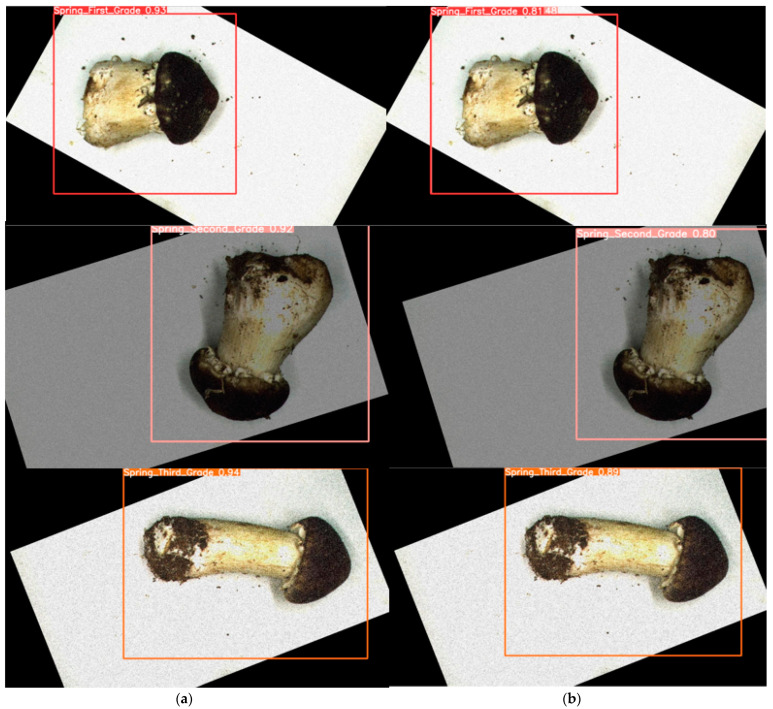
Detection results of student model (APHS-YOLO) and teacher model: (**a**) Teacher model. (**b**) Student model.

Figure 4a,b show the detection results for the teacher and student models, respectively. It is evident that the teacher model is significantly more accurate than the student model, i.e., APHS-YOLO, in assisted training.

For a given detection target, the recognition accuracy (values in Figure 4 indicate precision) of the teacher model exceeds that of the student model. Consequently, the teacher model is well positioned to facilitate the training of the student model, thereby enhancing the target detection accuracy of the latter.

## 4. Results and Discussion

### 4.1. Ablation Experiments

The purpose of the ablation experiments was to validate the performance of the added module by examining the effect of removing a component from the algorithm on the model’s performance. The ablation experiments conducted on both the APHS-YOLO model and the teacher model effectively demonstrated the effect of adding this module, providing a complete demonstration of APHS-YOLO’s excellent usability performance. The results of the ablation experiments for the student model and the teacher model are presented in Table 5 and Table 6.

The table shows the comparative results of the two ablation experiments described above. Both modules for the student model fusion produced excellent results for the model lightweighting, while the modules for the teacher model fusion had positive effects on improving the model’s accuracy. Specifically, for the APHS-YOLO student model, the HSFPN module reduced the model’s parameters by 40.6%. The CSPPC and AKConv modules further reduced the parameters by 15.6% and 3%, respectively. Additionally, the HSFPN module reduced the model’s memory footprint by 34%. The model size was reduced by 9%, and the CSPPC module further reduced it by 15.8%. Additionally, the AKConv module resulted in a 4% reduction in the model size. For the teacher model, which improved YOLOv8s, the CGA module improved the model recognition accuracy by 1.3% and the mAP 0.5:0.95 by 12%. The ACmix module further improved the model recognition accuracy by 0.6%.

The final results show that the improved APHS-YOLO model is significantly better than the original YOLOv8n model in terms of the lightweight effect; the optimized YOLOv8s teacher model is better than the YOLOv8s model in terms of the recognition efficiency. The final student model is able to achieve the excellent results of a 62.8% decrease in the number of parameters, and a 57.1% decrease in the model memory occupation, while the accuracy of the teacher model is also improved by 1.9 percentage points. Using the improved YOLOv8s teacher model to assist in training the student model, APHS-YOLO can successfully satisfy the requirement of the lightweight and high accuracy.

In general, the student model is the primary model used for training, while the instructor model is used to supplement training. Moreover, feature distillation requires that the network structure of the teacher model and the student model are basically the same, so we chose the YOLOv8 model with an HSFPN as the feature pyramid structure as the base model for the distillation training. In this paper, we use the lightweight APHS-YOLO model as the student model. The YOLOv8s model with the addition of the ACmix self-attention mechanism and the CGA joint group attention mechanism and the HSFPN feature pyramid mechanism is used as the teacher model. The comparison of the training results is shown in Table 7.

### 4.2. Model Training with Feature Distillation

**Figure 5:**
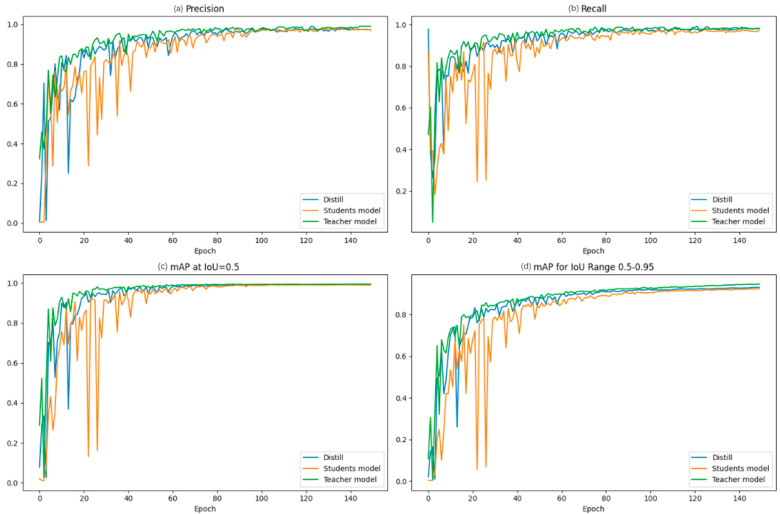
Comparison of metrics in models (student model, teacher model, distilled student model) related to knowledge distillation: (**a**) Precision curve. (**b**) Recall curve. (**c**) mAP@ 0.5 curve. (**d**) mAP@ 0.5:0.95 curve.

Figure 5 reflects the changes in the evaluation metrics with the number of training sessions during the model training process. From the following experiments, it can be seen that the model, after knowledge distillation, inherits the high recognition efficiency of the teacher model and maintains its own lightweight effect; in addition, the model, after feature refinement, converges faster than the original model, which is very obvious. This means that, compared with the existing methods for Stropharia rugoso-annulata recognition that only optimize in the direction of accuracy or lightweighting, APHS-YOLO can take both into account, and better complete the detection and classification tasks.

**Figure 6:**
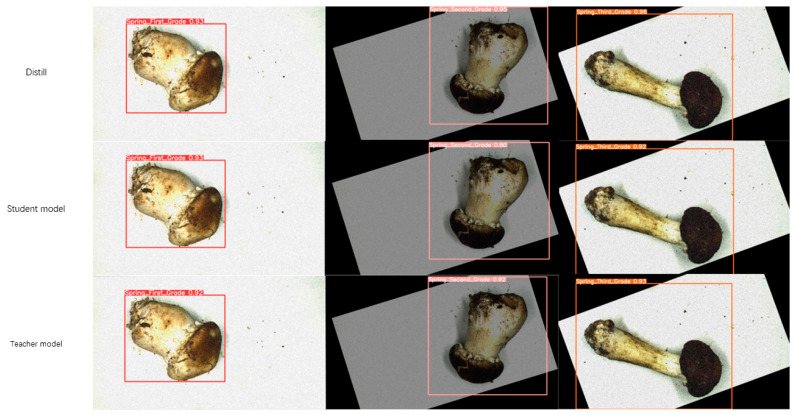
Comparison of three models of Stropharia rugoso-annulata grade detection after knowledge distillation.

Figure 6 demonstrates the excellent performance of APHS-YOLO on the task of identifying Stropharia rugoso-annulata grades after knowledge distillation training. It is clear that the APHS-YOLO model is far more effective than the other two base models in recognizing all three grades of Stropharia rugoso-annulata.

### 4.3. Visualization of Detection Results

**Figure 7:**
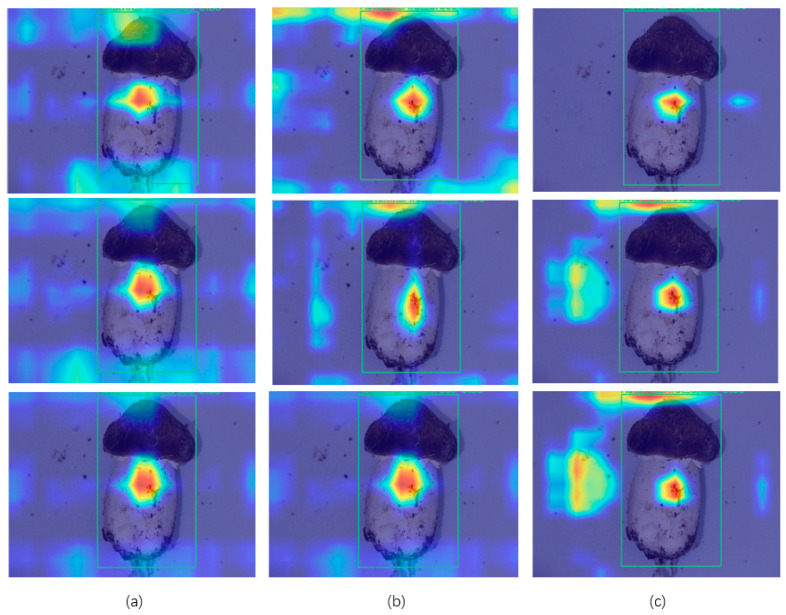
Heatmap of models related to knowledge distillation: (**a**) Student model. (**b**) Teacher model. (**c**) Distilled student model.

The change in value brought about by adding a module does not fully explain the effect of the module. As shown in Figure 7, in order to more clearly represent the effective facilitation of APHS-YOLO by the addition of the module, a heat map of the APHS-YOLO test results was generated to convert the complex data into a vivid color-coded matrix. This visualization tool uses a color spectrum to represent the different data values, with warmer shades indicating higher intensities and cooler shades indicating lower values. We can use the heatmap as a visual indicator to analyze the strengths and weaknesses of the module in extracting features and optimize APHS-YOLO in the future.

In a heat map, the more pronounced warm colors (e.g., red and yellow) indicate that the model allocates a greater degree of attention to the content of this part of the image when extracting features. Conversely, the more pronounced cooler colors (e.g., blue) receive less attention.

The heat map indicates that the models generated following knowledge distillation-assisted training tend to focus on feature inputs in the vicinity of Stropharia rugoso-annulata. This approach combines the strengths of both the teacher and student models, directing attention towards the Stropharia rugoso-annulata features while reducing the influence of irrelevant features from the student and teacher models.

### 4.4. Discussion

This study proposes the APHSYOLO algorithm for group classification and detection, based on the characteristics of the group dataset. Compared to existing algorithms, APHS-YOLO offers a high accuracy, fast detection speed, and light weight. It can be effectively deployed on devices with limited resources. The accuracy metrics exceeded those of the original model, with the FPS exceeding 100. The model size has been reduced by 58.7%, and the number of parameters has been significantly decreased. Based on the experimental graphs, APHS-YOLO demonstrates a faster convergence speed and higher accuracy rates compared to lighter models, with the assistance of teacher model training.

**Figure 8:**
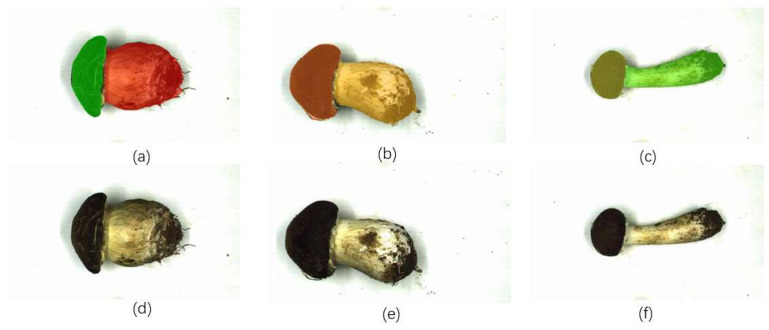
Image segment: (**a**) Segmented image of a first-class mushroom. (**b**) Segmented image of a second-class mushroom. (**c**) Segmented image of a third-class mushroom. (**d**) Original image of a first-class mushroom. (**e**) Original image of a second-class mushroom. (**f**) Original image of a third-class mushroom.

The current mushroom classification methods only address either the problems of accuracy or lightness. Therefore, these methods either have high accuracy but require sufficient device memory, which increases the cost, or are highly lightweight but difficult to control the accuracy of mushroom detection and classification. The classification method based on this network model has the following advantages over existing classification methods. The Stropharia rugoso-annulata classification method based on APHS-YOLO has a high recognition accuracy and can completely replace manual classification methods; secondly, the Stropharia rugoso-annulata classification method based on the APHS-YOLO model, because of innovative optimization at the algorithmic level, has a good lightweight performance and can be easily loaded onto devices with limited memory resources; third, existing non-manual Stropharia rugoso-annulata classification methods perform poorly under the working conditions of high-volume classification due to their detection speed problem, while the APHS-YOLO model-based classification method proposed in this study is carried out, taking into account the detection speed of the Stropharia rugoso-annulata, so that the class of Stropharia rugoso-annulata can be identified quickly for timely classification. This advantage means that the model does not require much equipment, and low-cost equipment can complete the high-precision identification, which can greatly reduce costs in industrialization. In addition, non-destructive testing with equipment to quickly and accurately classify can greatly improve the economic efficiency. However, it must be acknowledged that the model still has many areas requiring improvement and enhancement. A more comprehensive image processing method is needed to exploit the features of Stropharia rugoso-annulata. The method used in this paper is to directly annotate Stropharia rugoso-annulata images for identification. It was found that the growing conditions of Stropharia rugoso-annulata in the same harvesting season but in different grades may be difficult to distinguish, which may result in misclassification and affect the accuracy of recognition. In the future, we will establish a more accurate and fast intelligent grading method for Stropharia rugoso-annulata by segmenting the cap and stalk and exploring image feature quantification strategies. Figure 8 shows the preliminary design of the segmentation scheme.

## 5. Conclusions

An APHS-YOLO model is proposed for intelligent real-time grading of Stropharia rugoso-annulata as an alternative to manual grading. The model integrates the lightweight HSFPN neck module and AKConv convolution module, and the PConv module is optimized twice. A more lightweight module, the CSPPC module, is designed and used, and the knowledge distillation technique is applied to assist with the training of the APHS-YOLO model. APHS-YOLO is able to achieve the accurate identification of different seasons and grades of Stropharia rugoso-annulata under different light conditions and random environmental disturbances. While ensuring a high detection speed, APHS-YOLO significantly reduces the model volume, the number of model parameters, and the amount of model computation at the cost of a small reduction in the detection speed metrics, which enables the model to be efficiently deployed on resource-limited devices to accomplish accurate detection and grading tasks. Furthermore, the loss of accuracy metrics can be compensated for by knowledge distillation.

Innovations at the algorithmic level provide the possibility of the high-precision detection of Stropharia rugoso-annulata and the installation of the detection model on low-cost Stropharia rugoso-annulata sorting equipment, which is a prerequisite for the efficient real-time grading of Stropharia rugoso-annulata. The non-destructive detection method based on the APHS-YOLO model ensures the quality and food safety of Stropharia rugoso-annulata, and the intelligent real-time grading method based on the APHS-YOLO model replaces the traditional manual sorting method, which avoids the waste of manpower and material resources and reduces the cost of industrialization, and has excellent economic benefits.

In the future, we will continue to work on a lightweight feature extraction module to reduce the size of the model while increasing its detection speed, so as to extract features of Stropharia rugoso-annulata in different seasons and grades more efficiently. In addition, we plan to add Stropharia rugoso-annulata damage detection tasks to the grade detection and classification tasks to further safeguard food quality and safety issues in Stropharia rugoso-annulata in order to improve economic efficiency and promote the development of smart forestry and smart agriculture.

## Figures and Tables

**Table 1:** Grading criteria of Stropharia rugoso-annulata.

	Season	Spring	Autumn
Indicators			
0.0 < RDHP ≤ 2.5 0.0 < RLDS ≤ 3.0		First Grade	
2.5 < RDHP ≤ 4.0 3.0 < RLDS ≤ 4.5		Second Grade	
4.0 < RDHP 4.5 < RLDS		Third Grade	

Here, RDHP denotes the ratio of the diameter to the height of the cap, and RLDS denotes the ratio of the length to the diameter of the stalk.

**Table 2:** Partition of the dataset.

	Original	Enhanced
Number of train sets	1317	5268
Number of test sets	209	836
Number of valid sets	209	836

**Table 3:** Hyperparameter settings using in model training experiment.

Hyperparameter	Configuration
Optimizer	SGD
Batch Size	32
Epoch	150
Image Size	640 × 640
Learning Rate	0.01
Workers	8

**Table 4:** Detect training experiment with different types of YOLOv8 (Bolded data in black indicate the most significant effect in the indicator).

Model	Depth	Width	Parameters (M)	GFLOPs	FPS	*p*	mAP 0.5:0.95	Size (MB)
YOLOv8n	0.33	0.25	**3.2**	**8.1**	**188.0**	0.985	0.955	**6.3**
YOLOv8s	0.50	0.50	11.2	28.6	165.9	**0.994**	**0.972**	21.5
YOLOv8m	0.67	0.75	25.9	78.9	134.5	0.991	0.971	49.6
YOLOv8l	1.00	1.00	43.7	165.2	109	0.989	0.971	83.6
**YOLOv8x**	**1.00**	**1.25**	**68.2**	**257.8**	**104.3**	**0.993**	**0.972**	**130.4**

**Table 5:** Ablation experiments for student model (APHS-YOLO) (Bolded data in black indicate the most significant effect in the indicator).

YOLOv8n	HSFPN	CSPPC	AKConv	Parameters (M)	GFLOPs	FPS	*p*	mAP@ 0.5:0.95	Size (MB)
√	×	×	×	3.2	8.1	**188.0**	**0.985**	**0.955**	6.3 MB
√	√	×	×	1.9	6.9	175.4	0.972	0.946	4.1 MB
√	√	√	×	1.4	5.3	196.4	0.978	0.924	3.1 MB
√	√	√	√	**1.2**	**4.8**	112.7	0.976	0.922	**2.7 MB**

**Table 6:** Ablation experiments for teacher model (Bolded data in black indicate the most significant effect in the indicator).

YOLOv8s	HSFPN	CSPPC	CGAttention	ACmix	*p*	R	mAP@0.5	mAP @0.5:0.95
√	√	√	×	×	0.972	0.984	0.994	0.946
√	√	√	√	×	0.985	**0.99**	0.994	**0.958**
√	√	√	√	√	**0.991**	0.982	**0.995**	0.946

**Table 7:** Comparison between knowledge distillation model and two original models in terms of training results (Bolded data in black indicate the most significant effect in the indicator).

Model	Parameters (M)	GFLOPs	FPS	*p*	mAP@ 0.5:0.95	Size (MB)
Student model	1.2	4.8	112.7	0.976	0.922	2.7 MB
Teacher model	12.1	29.8	49.1	**0.994**	**0.963**	24.9 MB
Knowledge distillation model	**1.19**	**4.5**	**112.9**	0.980	0.931	**2.6 MB**

**Figure A4:**
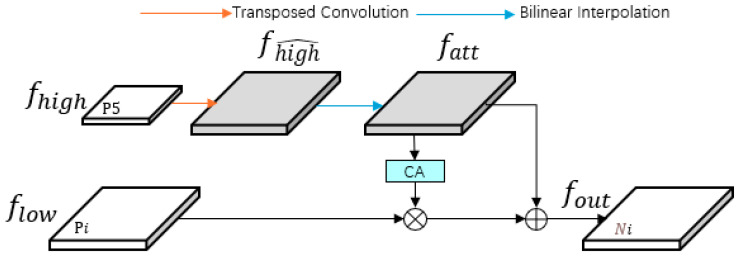
The framework of SFF module. The combination of transposed convolution and bilinear interpolation (Reprinted/adapted with permission from Ref. [11]. 2024, Ren-Ming Liu).

## Data Availability

The original contributions presented in the study are included in the article, further inquiries can be directed to the corresponding author.
